# A comprehensive re-analysis of the Golden Spike data: Towards a benchmark for differential expression methods

**DOI:** 10.1186/1471-2105-9-164

**Published:** 2008-03-26

**Authors:** Richard D Pearson

**Affiliations:** 1 School of Computer Science, University of Manchester, Oxford Road, Manchester, M13 9PL, UK

## Abstract

**Background:**

The Golden Spike data set has been used to validate a number of methods for summarizing Affymetrix data sets, sometimes with seemingly contradictory results. Much less use has been made of this data set to evaluate differential expression methods. It has been suggested that this data set should not be used for method comparison due to a number of inherent flaws.

**Results:**

We have used this data set in a comparison of methods which is far more extensive than any previous study. We outline six stages in the analysis pipeline where decisions need to be made, and show how the results of these decisions can lead to the apparently contradictory results previously found. We also show that, while flawed, this data set is still a useful tool for method comparison, particularly for identifying combinations of summarization and differential expression methods that are unlikely to perform well on real data sets. We describe a new benchmark, AffyDEComp, that can be used for such a comparison.

**Conclusion:**

We conclude with recommendations for preferred Affymetrix analysis tools, and for the development of future spike-in data sets.

## Background

The issue of method validation is of great importance to the microarray community; arguably more important than the development of new methods [1]. The microarray analyst is faced with a seemingly endless choice of methods, many of which give evidence to support their claims of being superior to other approaches, which at times can appear contradictory. Because of this, choice of methods is often determined not by a rigorous comparison of method performance, but by what a researcher is familiar with, what a researcher's colleagues have expertise in, or what was used in a researcher's favorite paper. Method validation is a difficult problem in microarray analysis because, for the vast majority of microarray data sets, we don't know what the "right answer" really is. For example, in a typical analysis of differential gene expression, we rarely know which genes are truly differentially expressed (DE) between different conditions. Perhaps even worse than this, we rarely have any strong evidence about the proportion of genes that are differentially expressed.

Perhaps the most well-known and widely used benchmark for Affymetrix analysis methods is Affycomp [2]. This is essentially a benchmark for normalization and summarization methods. While a very valuable tool of method validation, Affycomp is not ideal for comparison of DE methods because:

1. It uses data sets which only have a small number of DE spike-in probesets.

2. It only uses fold change (FC) as a metric for DE detection, and hence cannot be used to compare other competing DE methods.

More recently, the MicroArray Quality Control (MAQC) study [3] has developed a large number of reference data sets. The primary goal of this study was to show that microarray results can be reproducible, however, a secondary goal was to provide tools for benchmarking methods. The study concluded that using FC as a DE method gives results that are more reproducible than the other DE methods studied. However, the study could not give recommendations about other important metrics for DE methods such as sensitivity and specificity. The problem here is that we don't know for sure which genes are differentially expressed between the conditions. We could infer this by comparing results across the different microarray technologies used, but the different technologies may well have similar biases, invalidating the results. We could also infer which genes are differentially expressed by comparison with other technologies such as qRT-PCR, but again, there could be similar biases in these technologies. Furthermore, there are competing methods for detection of DE genes using qRT-PCR, so we may well get contradictory results when comparing different microarray DE methods against different qRT-PCR DE methods.

The "Golden Spike" data set of Choe *et al*. [4] includes two conditions; control (C) and sample (S), with 3 replicates per condition. Each array has 14,010 probesets. 3,866 of these probesets can be used to detect RNAs that have been spiked in. 2,535 of these spike-in probesets relate to RNAs that have been spiked-in at equal concentrations in the two conditions. The remaining 1,331 probesets relate to RNAs that have been spiked-in at higher concentrations in the S condition relative to the C condition. As such, this data set has a large number of probesets that are known to be DE, and a large number that are known to be not DE. This makes the Golden Spike data set potentially very valuable for validating DE methods.

There have been criticisms of the Golden Spike data set from Dabney and Storey [5], Irizarry *et al*. [6] and Gaile and Miecznikowski [7]. The main criticisms of [5] and [7] center around the fact that the non-DE probesets in the Golden Spike data set have non-uniform p-value distributions. This implies that any measure of significance of DE will be incorrect. Significance measures are valuable because they allow a researcher to make principled decisions about how many genes might be DE, which is a goal towards which we should strive. Unfortunately, we still have no way of knowing for sure whether the non-uniform p-value distributions of the non-DE probesets seen in the Golden Spike data set are particular to this data set, or are a general feature of microarray data sets. Indeed, a recent study by Fodor *et al*. [8] has suggested non-uniform p-value distributions may be common. However, even if we cannot reliably predict the proportion of genes that are differentially expressed, we can still rank the genes from most likely to be DE to least likely to be DE. In many cases, a researcher might want a list of candidate genes which will be investigated further. A common though admittedly unprincipled approach is to choose the top N candidate genes where N is determined by available resources rather than statistical significance. In such situations it is the rank order of probability of being DE that is used. The tool that has been used most extensively for comparing methods on this data set is the receiver-operator characteristic (ROC) chart. The ROC chart only takes into account the rank order of DE probesets, and hence is not affected by concerns about non-uniform p-value distributions. Gaile and Miecznikowski [7] show that the Golden Spike data set is not suitable for comparison of methods of false discovery rate (FDR) control, but say nothing about whether or not the data set can be used for comparing methods of ranking genes by propensity to be DE.

Irizarry *et al*. [6] detail three undesirable characteristics of the Golden Spike data set induced by the experimental design, and one artifact. The three undesirable characteristics are:

1. Spike-in concentrations are unrealistically high.

2. DE spike-ins are all one-way (up-regulated).

3. Nominal concentrations and FC sizes are confounded.

While we agree that these are indeed undesirable characteristics, and would recommend the creation of new spike-in data sets that do not have these characteristics, we do not believe that these completely invalidate the use of the Golden Spike data set as a useful comparison tool.

Perhaps more serious is the artifact identified by Irizarry *et al*. [6]. They show that the FCs of the spike-ins that are spiked in at equal levels are lower than the "empty" probesets (i.e. those not spiked in). Schuster *et al*. [9] have recently suggested that this difference is due to differences in non-specific binding, which in turn is due to differences in amounts of labeled cRNA between the C and S conditions. We agree that this artifact invalidates comparison methods that use the set of all unchanging (equal FC and empty) probesets as true negatives when creating ROC charts. However, we argue that we can still use the Golden Spike data set as a valid benchmark by using ROC charts with just the equal FC probesets as our true negatives (i.e. by ignoring the empty probesets).

The Golden Spike data set has been used to validate many different methods for summarizing Affymetrix data sets. Choe *et al*. [4] originally used this data set to show that a modified form of MAS5.0 (which we will refer to as CP for Choe Preferred) outperforms RMA [10], GCRMA [11] and MBEI (the algorithm used in the dChip software) [12]. Liu *et al*. [13] used the data set to show that multi-mgMOS [14] can outperform CP. Hochreiter *et al*. [15] used the data set to show that FARMS outperforms RMA, MAS5.0 and MBEI, and that RMA outperforms MAS5.0 and MBEI, in apparent contradiction to Choe *et al*. [4]. Chen *et al*. [16] used the data to show that DFW and GCRMA outperform RMA, MAS5.0, MBEI, PLIER [17], FARMS and CP, again in apparent contradiction to Choe *et al*. [4]. All of these papers used some form of ROC curve in their analyses. The confusing, and seemingly contradictory results, make it difficult for typical Affymetrix users to decide between methods.

The reason for the differing results arise from the different choices made at various stages of the analysis pipeline. In particular, different DE methods have been used in the papers cited above. Only Choe *et al*. [4] and Liu *et al*. [13] have compared different DE methods on the results of the same normalization and summarization methods. Choices for DE methods include: fold change (FC); t-tests; modified t-tests such as those used by limma [18] and Cyber-T [19]; and the probability of positive log ratio (PPLR) method [13]. In addition to choice of DE method, there are choices to be made at other stages of the analysis pipeline. We broadly summarize these as the following six choices, each of which can have a significant influence over results:

1. Summary statistic used (e.g. RMA, GCRMA, MAS5.0, etc.). Note that Choe *et al*. [4] broke this particular choice down to four separate sub-choices of methods for background correction, probe-level normalization, PM adjustment, and expression summary.

2. Post-summarization normalization method. Choe *et al*. [4] compared no further normalization against the use of a loess probeset-level normalization based on the known invariant probesets.

3. Differential expression (DE) method. Choe *et al*. [4] compared t-test, Cyber-T [19] and SAM [20].

4. Direction of differential expression. Choe *et al*. [4] used a 2-sided test (as opposed to, for example, a 1-sided test of up-regulation).

5. Choice of true positives. Choe *et al*. [4] used all spike-in probesets with fold-change (FC) greater than 1.

6. Choice of true negatives. Choe *et al*. [4] used all invariant probesets. This included both probesets that were spiked in at equal quantities, as well as the so-called "empty" probesets.

Table 1 shows the choices we believe were made in various studies of the Golden Spike data set. In addition to the studies identified in Table 1, Lemieux [21] and Hess and Iyer [22] report results of "probe-level" methods for detecting differential expression. We do not consider these approaches here. In addition to the choices at the six steps of the analysis pipeline highlighted above, there are choices to be made about how the data are displayed, and what metrics should be used for comparison. There are many types of "ROC-like" charts that can be created. An ROC chart is generally considered to be one where the x-axis shows the false-positive rate (FPR), and the y-axis the true-positive rate (TPR). This type of chart is used in the Liu *et al*. [13], Hochreiter *et al*. [15] and Chen *et al*. [16] papers. Another type of ROC curve has the false-discovery rate (FDR) along the x-axis. This type of ROC curve was used in the original Choe *et al*. paper [4]. There are a large range of other types of chart for visualizing classifier performance [23] that we have not considered. In addition, choices need to be made about whether to show the full ROC charts (with x- and y-axes both between 0 and 1), or whether to just display a part of the chart. While using the full ROC chart is the only way of assessing the performance of a method across the full range of data, this can result in charts where the lines of each method are very close together and hence difficult to distinguish. Often, an analyst is most interested in methods which will give the least number of false positives for a relatively small number of true positives, as only a small number of genes will be investigated further. In such cases it can often be informative to show the ROC chart for a much smaller range of FPRs, for example, between 0 and 0.05. The charts in the original Choe *et al*. paper [4] use different x-axis cutoffs to show different aspects of the analysis.

**Table 1:** Analysis choices of various studies of the "Golden Spike" data set. These are choices we believe were made for each of the six stages of the analysis pipeline we have outlined.

Study	Summarization method	Post-summ Normalization	DE method	Dir	True positives	True negatives
Choe *et al*. [4]	CP, MAS5.0, RMA, GCRMA, MBEI plus many variants of these	none, loess_invariant	t-test, Cyber-T, SAM	either	FC >1	invariant
Liu *et al*. [13]	CP, multi-mgMOS	loess_invariant	Cyber-T, PPLR	up	FC >1	invariant
Hochreiter *et al*. [15]	MAS5.0, RMA, MBEI and FARMS	none	SAM	up	FC >1	invariant
Chen *et al*. [16]	CP, MAS5.0, RMA, GCRMA, MBEI, PLIER, FARMS and DFW	none	FC	either	FC >1 and FC = x(for all x)	invariant
Current study	CP, MAS5.0, RMA, GCRMA, MBEI, multi-mgMOS, FARMS, DFW, PLIER	none, loess_invariant, loess_equal, loess_all	FC, t-test, Cyber-T, limma and PPLR	either, up and down	FC >1, low FC, medium FC, high FC and FC = x(for all x)	equal and invariant

The most commonly used metric for assessing a DE detection method's performance is the Area Under the standard ROC Curve (AUC). This is typically calculated for the full ROC chart (i.e. FPR values from 0 to 1), but can also be calculated for a small portion of the chart (e.g. FPRs between 0 and 0.05). Other metrics that might be used are the number or proportion of true positives for a fixed number or proportion of false positives, or conversely the number or proportion of false positives for a fixed number or proportion of true positives.

In this study we have analyzed all combinations of the various options shown in the last row of Table 1. In addition, we have created charts displaying the data in different ways. In the next section we show how results can vary when making different choices at the stages of the analysis pipeline highlighted above. We also discuss what we believe are good choices. We detail a web resource called AffyDEComp which can be used as a limited benchmark for DE methods on Affymetrix data. We also highlight some issues of reproducibility in comparative studies. We conclude by making recommendations on choices of Affymetrix analysis methods, and desired characteristics of future spike-in data sets.

## Results and Discussion

### Direction of Differential Expression

**Figure 1:**
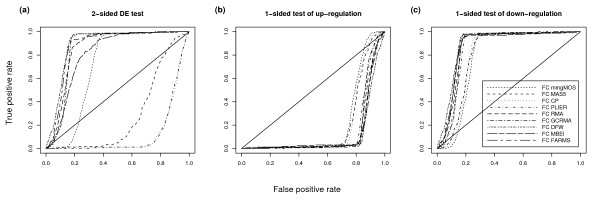
**Comparison of 1- and 2-sided tests of DE for very low FC genes**. ROC charts of Golden Spike data using a 2-sided and two 1-sided tests of DE. For these charts all unchanging probesets are used as true negatives, genes with FC of 1.2 are used as true positives, and no post-summarization normalization is used. We only show results for the FC DE detection method. The different charts show a.) probesets selected using a 2-sided test of DE, b.) probesets selected using a 1-sided test of up-regulation and c.) probesets selected using a 1-sided test of down-regulation. The diagonal line shows the "line of no-discrimination". This shows how well we would expect random guessing of class labels to perform.

We can see from Table 1 that studies to date have used either a 1-sided test or a 2-sided test for differential expression. A potential problem with using a 2-sided test on this data set becomes apparent if we compare the tests using the other analysis choices of Chen *et al*. [16]. Figure 1 shows the ROC charts created using a 2-sided test of differential expression, and 1-sided tests of up- and down-regulation. This was created using just those probesets that have a FC of 1.2 as true positives. Figure 1a is the equivalent of Figure 3 of Chen *et al*. [16]. This appears to show that DFW has the strongest performance. However, if we look at Figure 1b and Figure 1c we see that the methods that appear to be performing strongly in Figure 1a are actually mainly detecting down-regulated genes. The reason for this becomes clear when we look at Figure 2 from Irizarry *et al*. [6]. There we see that spike-in genes with small fold changes greater than 1, actually have M values (i.e. fold changes) generally less than the M values of the "empty probesets" which form the majority of the negatives from which this chart was created.

The choice of whether 1-sided or 2-sided tests should be used for comparison of methods is debatable. A 1-sided test for down-regulation is clearly not a sensible choice given that all the known DE genes are up-regulated. We would expect a 1-sided test of up-regulation to give the strongest results, given that all the unequal spike-ins are up-regulated. However, in most real microarray data sets, we are likely to be interested in genes which show the highest likelihood of being DE, regardless of the direction of change. As such, we will continue to use both a 2-sided test, and a 1-sided test of up-regulation in the remainder of the paper. In our comprehensive analysis, however, we also include results for 1-sided tests of down-regulation for completeness.

### True negatives

**Figure 2:**
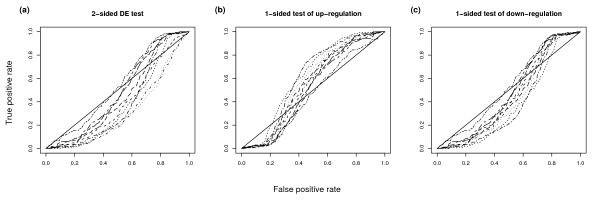
**Comparison of 1- and 2-sided tests using only equal spike-ins as true negatives**. ROC charts of Golden Spike data using a 2-sided and two 1-sided tests of DE, with only the equal spike-ins used as true negatives. Genes with FC of 1.2 are used as true positives, and no post-summarization normalization is used. We only show results for the FC DE detection method. The legend is the same as in Figure 1. The different charts show a.) probesets selected using a 2-sided test of DE, b.) probesets selected using a 1-sided test of up-regulation and c.) probesets selected using a 1-sided test of down-regulation. As with Figure 1, we include lines of no-discrimination.

Figure 2 shows the ROC charts created using the same choices as used in Figure 1, except that this time we use just the probesets which have been spiked in at equal concentrations as our true negatives. Here we see a very different picture. Firstly, the differences between different summarization methods are less pronounced when using a 2-sided test of DE. Also, the charts for detecting up- and down-regulated genes are quite similar. This indicates that it is actually very difficult for methods to distinguish these two classes. This is perhaps not surprising given the similarities in the fold changes (the true negatives have a FC of 1 and the true positives have a FC of 1.2). We should note, however, that the ROC curves detecting up-regulation (Figure 2b) are generally slightly above the diagonal (i.e. slightly better than random guessing), whereas the ROC curves detecting down-regulation (Figure 2c) are generally slightly below the diagonal (i.e. slightly worse than random guessing). This gives us confidence that by just using equal-valued spike-ins as our true negatives, our ROC curves can detect genuine improvements in detecting DE genes due to different methods.

**Figure 3:**
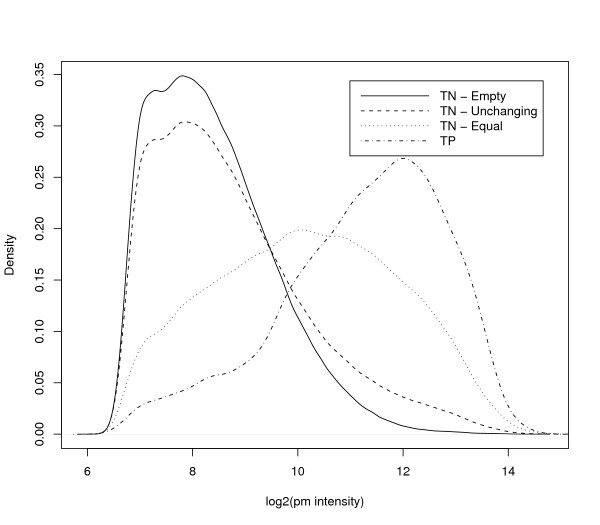
**Density plots of intensities for different choices of true negatives**. These plots show the distributions of intensities of perfect match (PM) probes across all six arrays of the Golden Spike data, for different subsets of probesets. We show plots for three potential choices of true negative (TN) probesets: the Empty probesets are defined as those for which there is no corresponding spike-in RNAs. The Equal probesets are defined as those spiked in at equal concentrations in the C and S conditions. The Unchanging probesets are defined as the set of all Empty and Equal probesets. For this chart we have defined true positives (TP) as those probesets which have been spiked in at higher concentration in the S condition relative to the C condition.

Irizarry *et al*. [6] showed that the FCs of the equal concentration spike-ins are quite different from those of the empty probesets. Another difference between these two sets of probesets is in their intensities. Figure 3 shows density plots of the intensities of the equal and empty probesets. Figure 3 also shows density plots of intensities of unchanging (i.e. equal or empty) probesets, and of the true positives (spike-ins with FC > 1). The first thing to note is that the plots for empty and unchanging probesets are very similar. This is to be expected as there are many more empty probesets than equal probesets. We also see that, although there are differences between the equal and TP plots (the confounding between concentration and FC identified by Irizarry *et al*. [6]), these are not nearly so pronounced as the differences between the unchanging and TP plots. Indeed, from Figure 3 we can see that a classifier based purely on intensity alone would separate well the unchanging probesets from the TPs. This fact, together with the artifact identified by Irizarry *et al*. [6], leads us to recommend using only the equal concentration spike-ins as the set of true negatives for method comparison. In our comprehensive analysis, however, we also include results when using all the unchanging probesets, for completeness.

### Post-Summarization Normalization

**Figure 4:**
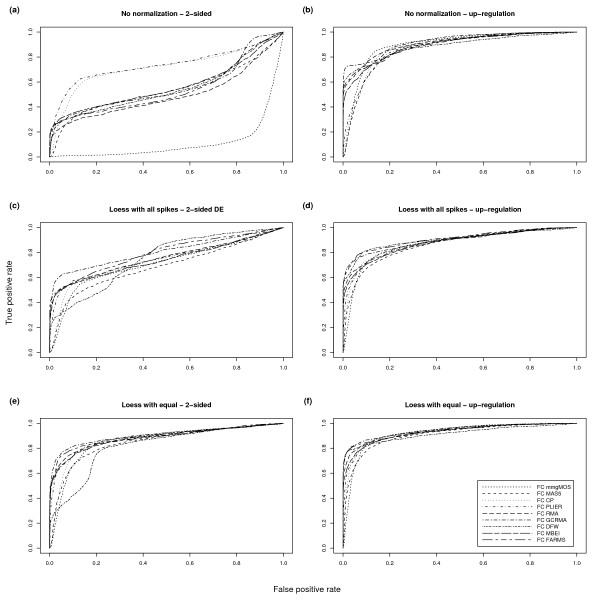
**Comparison of different post-summarization normalization strategies**. ROC charts of Golden Spike data using a 2-sided and a 1-sided test of DE, and using three different post-summarization normalization strategies. For these charts only the equal spike-ins are used as true negatives, and all spike-ins with FC > 1 are used as true positives. We only show results for the FC DE detection method. The top row relates to data sets created without any post-summarization normalization. The middle row relates to data sets created using all probesets for the loess normalization. The bottom row relates to data sets created using only the equal spike-in probesets for the loess normalization. The left column shows probesets selected using a 2-sided test of DE. The right column shows probesets selected using a 1-sided test of up-regulation.

Thus far, we have not considered the effect of post-summarization normalization, which was shown by Choe *et al*. [4] to have a significant effect on results. Figure 4 shows the effect of such normalizations. Note that unlike Figures 1 and 2 we are here treating all of the spike-ins with FC > 1 as our true positives, not just those with FC = 1.2. Here we can see that post-summarization loess normalization improves results, which is consistent with the results of Choe *et al*. [4]. Furthermore, we see that post-summarization normalization using just the equal-valued spike-ins improves results to a greater extent than using a loess normalization based on all probesets.

We agree with Gaile and Miecznikowski [7] that "the invariant set of genes used for the pre-processing steps in Choe *et al*. should not have included the empty null probesets". As such, for the remainder of this paper will we not use the empty probesets in loess normalization. In our comprehensive analysis we also include, for completeness, results when using all of the following post-summarization normalization strategies: no post-summarization normalization, a loess normalization based on all spike-in probesets, a loess normalization based on all the unchanging probesets and a loess normalization based on the equal-valued spike-ins.

### Differential Expression Detection Methods

**Figure 5:**
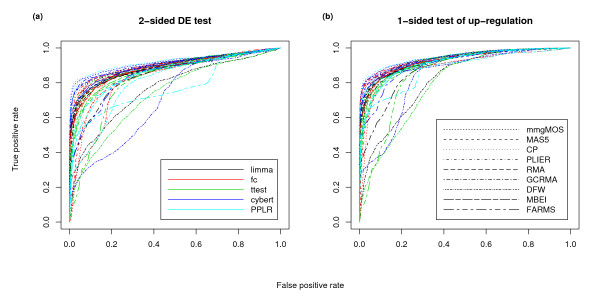
**Comparison of combinations of summarization/DE detection methods**. ROC charts of Golden Spike data using a 2-sided and a 1-sided test of DE, using different combinations of summarization and DE detection methods. For these charts only the equal spike-ins are used as true negatives, and all spike-ins with FC > 1 are used as true positives. A post-summarization loess normalization based on the equal-valued spike-ins was used. The different charts show a.) probesets selected using a 2-sided test of DE, and b.) probesets selected using a 1-sided test of up-regulation. The two legends refer to both a.) and b.)

We turn now to the issue of DE detection methods. Figure 5 shows ROC charts created with different combinations of summarization and DE methods. Different colors are used to identify different DE methods, and different line types are used to identify different summarization methods. Tables 2 and 3 show the AUCs of the ROC charts of Figure 5, with the top 10 performing combinations of summarization and DE detection methods shown in bold. Of the DE methods, Cyber-T appears to have particularly good performance, with 5 of the top 10 AUCs when using a 2-sided test, and 4 of the top 10 AUCs when looking specifically for up-regulation. Of the other DE methods, limma is the only method to have more than 1 AUC in the top 10 for both 2-sided and 1-sided tests. Looking at the summarization methods, multi-mgMOS has 4 AUCs in the top 10 for both 2-sided and 1-sided tests, while both CP and GCRMA have 2 AUCs in the top 10 for both tests. The top AUC in both 2-sided and 1-sided tests is obtained using multi-mgMOS and PPLR.

**Table 2:** AUCs for 2-sided test of DE. This table shows AUC values for different combinations of summarization and DE detection methods. The 10 highest AUC values are highlighted in bold. Note that the PPLR method is only applicable to summarization methods that give uncertainty estimates as well as mean expression levels for each probeset. These results were calculated using only the equal spike-ins as true negatives, and all spike-ins with FC > 1 as true positives. A post-summarization loess normalization using the equal-valued spike-ins was used. The results in this table are for 2-sided tests of DE.

	limma	FC	t-test	Cyber-T	PPLR
mmgMOS	**0.903**	0.861	**0.902**	**0.919**	**0.922**
MAS5	0.884	0.848	0.879	**0.905**	
CP	**0.905**	0.873	0.898	**0.919**	0.889
PLIER	0.898	0.889	0.889	**0.911**	
RMA	0.881	0.885	0.858	0.886	0.860
GCRMA	0.890	**0.902**	0.883	**0.909**	
DFW	0.764	0.815	0.732	0.703	0.806
MBEI	0.885	0.884	0.870	0.897	0.855
FARMS	0.842	0.891	0.805	0.844	0.772

**Table 3:** AUCs for 1-sided test of up-regulation. This table shows AUC values for different combinations of summarization and DE detection methods. The 10 highest AUC values are highlighted in bold. Note that the PPLR method is only applicable to summarization methods that give uncertainty estimates as well as mean expression levels for each probeset. These results were calculated using only the equal spike-ins as true negatives, and all spike-ins with FC > 1 as true positives. A post-summarization loess normalization using the equal-valued spike-ins was used. The results in this table are for 1-sided tests of up-regulation.

	limma	FC	t-test	Cyber-T	PPLR
mmgMOS	**0.940**	0.920	**0.938**	**0.949**	**0.951**
MAS5	0.924	0.908	0.921	0.934	
CP	**0.940**	0.928	0.935	**0.948**	0.932
PLIER	0.934	0.929	0.930	**0.941**	
RMA	0.929	0.932	0.914	0.932	0.917
GCRMA	0.926	**0.946**	0.921	**0.944**	
DFW	0.817	0.918	0.794	0.830	0.912
MBEI	0.928	0.928	0.920	0.934	0.915
FARMS	0.883	**0.938**	0.847	0.908	0.893

**Figure 6:**
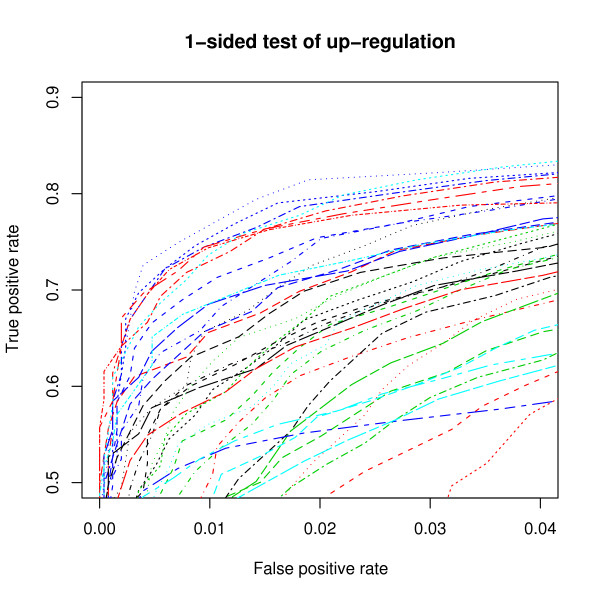
**Comparison of combinations of summarization/DE detection methods at low false positive rates**. ROC charts of Golden Spike data using a 1-sided test of DE, using different combinations of summarization and DE detection methods, and showing only false positive rates between 0 and 0.04, and false negative rates between 0.5 and 0.9. For these charts only the equal spike-ins are used as true negatives, and all spike-ins with FC > 1 are used as true positives. A post-summarization loess normalization based on the equal-valued spike-ins was used. The legend is the same as in Figure 5.

The end goal of an analysis is often to identify a small number of genes for further analysis. As such, we might be interested not in how well a method performs on the whole of a data set, but specifically in how well it performs in identifying those genes determined to be most likely to be DE. As such we are particularly interested in the ROC chart at the lowest values of FPR. Figure 6 shows the same ROC curves as Figure 5b up to FPR values of 0.04. From Figure 6 we can see that, although the combination of multi-mgMOS and PPLR has the highest overall AUC, this method does not have the strongest performance for most values of FPR between 0 and 0.04. For FPR values between about 0.005 and 0.03, the combination of CP and Cyber-T has the strongest performance. For even lower FPR values, both FARMS and DFW in combination with FC are the strongest performers for small ranges of FPR.

Figure 6 can be used for overall comparisons of DE methods. In general, we see that Cyber-T tends to outperform limma, and both of these methods generally outperform the use of standard t-tests. The performance of FC as a DE detection method varies much more, depending on the summarization method used. When FC is used in combination with DFW, FARMS or GCRMA, performance is generally amongst the best. However, performance of FC with RMA, MBEI and PLIER is less strong, and the combination of FC with multi-mgMOS, MAS5.0 or CP is particularly poor. Of the summarization methods that perform well with FC, FARMS and DFW have generally poor performance when used in combination with other methods. GCRMA has reasonable performance in combination with Cyber-T, but is in the lower half of summarization methods when used in combination with either limma or standard t-tests.

### True positives

So far we have used all of the genes that are spiked-in at higher concentrations in the S samples relative to the C samples as our true positives. This is perhaps the best and fairest way to determine overall performance of a DE detection method. However, we might also be interested in whether certain methods perform particularly well in "easier" or "more difficult" cases. Indeed, many analysts are only interested in genes which are determined not only to have a probability of being DE that is significant, but also have a FC which is greater than some pre-determined threshold. In order to determine which methods perform more strongly in "easy" or "difficult" cases, we can restrict our true positives to just those genes than are known to be DE by just a small FC, or to those that are very highly DE.

**Figure 7:**
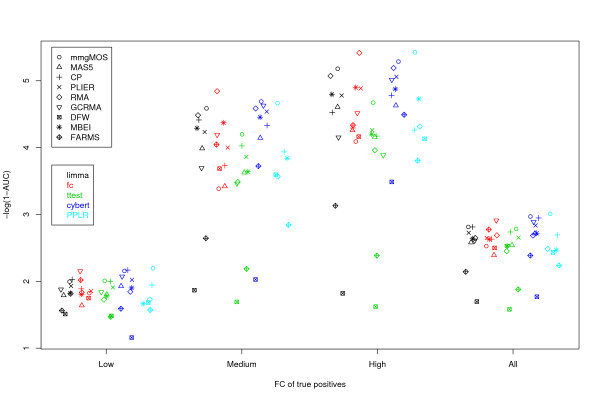
**Comparison of different choices of true positives**. Areas under ROC curves of Golden Spike data using different combinations of summarization and DE detection methods, and different sets of true positives. For these charts only the equal spike-ins are used as true negatives. The chart shows probesets selected using a 1-sided test of up-regulation. The Low true positives are those spike-ins with a FC greater than 1 but less than or equal to 1.7. The Medium true positives are those spike-ins with a FC between 2 and 2.5 inclusive. The High true positives are those spike-ins with a FC greater than or equal to 3. The y-axis shows -log(1-AUC) rather than AUC, as this gives a better separation between the higher AUC values, but retains the same rank order of methods. The x-axis is categorical, with points jittered to avoid placement on top of each other.

Figure 7 shows AUC values where the true positives are a subset of all the DE genes. The subsets are determined by the known FCs. The first thing to note from Figure 7 is that methods generally perform much better at detecting high FC genes, than they do in detecting low FC genes. This is to be expected of course. From Figure 7 we can also see that methods that perform well overall tend to also perform well regardless of whether the FCs are low, medium or high. There are, nonetheless, differences in the ranking of methods in each case. For example, although the combination of multi-mgMOS and PPLR was shown to have the highest AUC overall, it is outperformed by the combination of RMA and FC when considering either medium or high FC genes as true positives. Conversely, RMA/FC is outperformed by many other summarization/DE detection combinations for low FC genes. These results show us that the performance of a method may depend on the balance of easy and difficult cases.

### Comprehensive Analysis

We have created ROC charts for each combination of analysis choices from the final row of Table 1. For each of these combinations we have created ROC charts where the x-axis shows FPR, and where the x-axis shows FDR. We have also created charts where FPR/FDR has the full range of 0 to 1, and where FPR/FDR has the range 0 to 0.05. We have created a web resource called AffyDEComp [24] where ROC charts can be displayed by specifying the analysis pipeline choices. In addition, AUC charts similar to Figure 7 are also shown for different combinations of analysis pipeline choices. AffyDEComp also includes a table of thirteen key performance metrics for each combination of summarization and DE detection methods. The metrics used are:

1. AUC where equal-valued spike-ins are used as true negatives, spike-ins with FC > 1 are used as true positives, a post-summarization loess normalization based on the equal-valued spike-ins is used, and a 1-sided test of up-regulation is the DE metric. This gives the values shown in Table 3.

2. as 1. but using a 2-sided test of DE. This gives the values shown in Table 2.

3. as 1. but with low FC spike-ins used as true positives. This gives the values shown in Figure 7.

4. as 1. but with medium FC spike-ins used as true positives. This gives the values shown in Figure 7.

5. as 1. but with high FC spike-ins used as true positives. This gives the values shown in Figure 7.

6. as 1. but with all unchanging probesets used as true negatives.

7. as 1. but with all unchanging probesets used as true negatives, and a post-summarization loess normalization based on the unchanging probesets.

8. as 1. but with a post-summarization loess normalization based on all spike-in probesets.

9. as 1. but with a no post-summarization normalization.

10. as 1. but giving the AUC for FPRs up to 0.01.

11. the proportion of true positives without any false positives (i.e. the TPR for a FPR of 0), using the same conditions as 1.

12. the TPR for a FPR of 0.5, using the same conditions as 1.

13. the FPR for a TPR of 0.5, using the same conditions as 1.

We are happy to include other methods if they are made available through Bioconductor packages. We also intend to extend AffyDEComp to include future spike-in data sets as they become available. In this way we expect this web resource to become a valuable tool in comparing the performance of both summarization and DE detection methods.

### Reproducible Research

One of the main problems with comparing different analyses of the same data sets is knowing exactly what code has been used to create results. As an example, the loess normalization used in a number of the papers shown in Table 1 has a "span" parameter. None of the papers mention what value has been used for this parameter, though Choe *et al*. [4] have made all their source code available, albeit on their website rather than as supplementary information to their paper. We believe that the only way to provide analysis results that are reproducible is to either:

1. provide full details of all parameter choices used in the papers Methods section, or

2. make the code used to create the results available, ideally as supplementary information to ensure a permanent record.

We recommend that journals should not accept method comparison papers unless either of these is done. This paper was prepared as a "Sweave" document [25]. The source code for this document is a mixture of LaTeX and R code. We have made the source code available as Additional file 1. This means that all the code used to create all the results in this paper, and in AffyDEComp [24], are available and all results can be recreated using open source tools.

## Conclusion

We have performed the most comprehensive analysis to date of the Golden Spike data set. In doing so we have identified six stages in the analysis pipeline where choices need to be made. We have made firm recommendations about the choices that should be made for just one of these stages if using the Golden Spike data for comparison of summarization and DE expression detection methods using ROC curves: we recommend that only the probesets that have been spiked-in should be used as the true negatives for the ROC curves. By doing this we overcome the problems due to the artifact identified by Irizarry *et al*. [6]. We would also recommend the following choices:

1. The use of a post-summarization loess normalization, with the equal spike-in probesets used as the subset to normalize with. This is also recommended by Gaile and Miecznikowski [7].

2. The use of a 1-sided test for up-regulation of genes between the C and S conditions. This mimics the actual situation because all the non-equal spike-ins are up-regulated.

3. The use of all up-regulated probesets as the true positives for the ROC chart.

Using the above recommendations, we created ROC charts for all combinations of summarization and DE methods (Figure 5b and Table 3). This showed us that there was no clear DE detection method that stood out, but that what is important is the combination of summarization and DE method. We saw that the combination of multi-mgMOS and PPLR gave the largest AUC. One of the downsides with the PPLR approach is that there is no principled way of determining the proportion of genes that are DE, as is claimed by some FDR methods. Other combinations that had strong performance included GCRMA/FC, and Cyber-T used in conjunction with various normalization methods. By looking at very small FPRs (Figure 6), CP/Cyber-T, FARMS/FC and DFW/FC were all shown to be potentially valuable when identifying a small number of potential targets. If looking only for genes with larger FCs (Figure 7), RMA/FC was seen to give the strongest performance.

It should be noted that the design of this experiment could favor certain methods. We have seen that the intensities of the spike-in probesets are particularly high. Estimates of expression levels are known to be more accurate for high intensity probesets. This could favor the FC method of determining DE.

Furthermore, the replicates in the Golden Spike study are technical rather than biological, and hence the variability between arrays might be expected to be lower in this data set than in a typical data set. Again, this might favor the FC DE method.

We agree with Irizarry *et al*. [6] that the Golden Spike data set is flawed. In particular, we recognize that in creating ROC charts from just those probesets which were spiked-in, we are using a data set where the probe intensities are higher than in many typical microarray data sets. Also, applying a post-summarization normalization is not something that many typical analysts will perform, but is believed to be necessary to overcome some of the limitations of this data set, namely that the experiment is unbalanced due to the fact that all the DE spike-ins are up-regulated. We believe that using only the equal-valued spike-in probesets, both as true negatives and for the post-summarization normalization, is the most appropriate way of analyzing this particular data set. Furthermore, given the issues highlighted in the introduction regarding Affycomp and comparisons with qRT-PCR results, we believe that the Golden Spike data set is still the most appropriate tool for comparing DE methods. To this end we have created the AffyDEComp benchmark to enable researchers to compare DE methods. However, we should stress that we are not, at this stage, recommending that AffyDEComp be used as a reliable benchmark as the Golden Spike data set might not be representative of data sets more generally. In particular, just because a method does well here, doesn't necessarily mean that the method will do well generally. At this time, AffyDEComp might better be suited to identifying combinations of summarization and DE detection methods that perform particularly poorly.

We encourage the community to develop further spike-in data sets with large numbers of DE probesets. In particular, we encourage the generation of data sets where:

1. Spike-in concentrations are realistic

2. DE spike-ins are a mixture of up- and down-regulated

3. Nominal concentrations and FC sizes are not confounded

4. The number of arrays used is large enough to be representative of some of the larger studies being performed today

We believe that only by creating such data sets will we be able to ascertain whether the artifact noted by Irizarry *et al*. [6] is a peculiarity of the Golden Spike data set, or is a general feature of spike-in data sets. More importantly, the creation of such data sets should improve the AffyDEComp benchmark, and hence enable the community to better evaluate DE detection methods for Affymetrix data.

## Methods

The raw data from the Choe *et al*. [4] study was originally downloaded from the author's website [26]. All analysis was carried out using the R language (version 2.6.0). MAS5.0, CP, RMA and MBEI expression measures were created using the Bioconductor [27]* affy *package (version 1.16.0). GCRMA expression measures were created using the Bioconductor *gcrma *package (version 2.10.0). PLIER expression measures were created using the Bioconductor *plier *package (version 1.8.0). multi-mgMOS expression measures were created using the Bioconductor *puma *package (version 1.4.1). FARMS expression measures were created using the *FARMS *package (version 1.1.1) from the author's website [28]. DFW expression measures were created using the *affy *package and code from the author's website [29]. Cyber-T results and Loess normalization were obtained using the *goldenspike *package (version 0.4) [26]. All other analysis was carried out using the Bioconductor *puma *package (version 1.4.1).

The code used to create all results in this document is included as Additional file 1.

## List of abbreviations

DE – differentially expressed or differential expression, as appropriate. FC – fold change. MAQC -MicroArray Quality Control. ROC – receiver-operator characteristic. FPR – false-positive rate. TPR -true-positive rate. FDR – false-discovery rate. AUC – area under curve (in this paper this refers to the area under the ROC curve).

## Authors' contributions

RDP designed the study, performed all analysis, developed the AffyDEComp website, and wrote the manuscript.

## Supplementary Material

Additional file 1 **Source code used to create this paper and AffyDEComp**. This is a zip file containing R and Sweave code. Sweave code is a text document which contains both LaTeX and R code, and as such can be used to recreate exactly all the results in this paper using open source tools. Also included is R code to recreate all the charts available through AffyDEComp. See the README file for further details. Click here for file
